# Increased nuchal translucency in fetuses with a normal karyotype—diagnosis and management: An observational study

**DOI:** 10.1097/MD.0000000000007521

**Published:** 2017-07-21

**Authors:** Demetra Socolov, Razvan Socolov, Vlad Eusebiu Gorduza, Tudor Butureanu, Ruxandra Stanculescu, Alexandru Carauleanu, Ioana Pavaleanu

**Affiliations:** a Department of Mother and Child Medicine, University of Medicine and Pharmacy Gr. T. Popa Iasi; b Hospital of Obstetrics and Gynecology Elena Doamna-Iasi; c Human Genetics Department, University of Medicine and Pharmacy Gr. T. Popa Iasi; d University of Medicine and Pharmacy Carol Davila-Bucharest, Romania.

**Keywords:** fetal nuchal translucency, karyotype markers, prenatal diagnostic

## Abstract

The use of nuchal translucency (NT) in 1992 by Nicolaides et al was a major breakthrough in screening for chromosomal aneuploidies at the end of the first trimester. However, pathological conditions other than chromosomal aneuploidies are also associated with increased NT, which can also be detected in normal fetuses. This study sought to evaluate the causes of this ultrasound sign in a group of patients from Iasi, Romania.

During the decade-long study period, there were 71 certified cases involving increased NT; the patients in these cases underwent diagnostic amniocentesis and karyotyping.

In most of the examined cases (55 cases, 78%), there was no aneuploidy. The remaining cases involved trisomy 21 (T21) (18%), trisomy 18 (T18) (2%), or triploidy (2%). In most cases, the indication for amniocentesis was increased NT alone (81%), whereas the remaining cases also involved advanced maternal age (5.5%), abnormal serologic markers (10%), or other ultrasound signs (3.5%) (2 cases—cardiac anomalies and fetal hydrops). A favorable pregnancy outcome at term was achieved in 40 cases (56% from total, 72% from euploid pregnancies); kidney anomalies or nonlethal cardiac conditions were observed in 12 cases (17%), 6 of which involved complications associated with premature onset of labor, and miscarriages occurred in 6 cases. Three cases were lost at follow-up.

Although it is common practice to assume that increased NT is an indication for amniocentesis, both literature results and our study findings indicate that the majority of cases with increased NT involve no aneuploidy and result in a favorable outcome if no other anomaly is present. Better evidence-based management of such cases could be promoted by conducting larger, multicenter studies, and following cases for longer periods.

## 1 Introduction

Although the thickened skin of Down syndrome fetuses was first mentioned in Langdon Down's initial description of such fetuses in 1866, only the development of ultrasound scanning allowed specialists to detect the nuchal edema presented by certain fetuses, as reported by Nicolaides in 1992. Once a standardized assessment to measure nuchal translucency (NT) was developed,^[1]^ an increasing number of reports substantiated the value of this ultrasound marker for screening for aneuploidy during the first trimester. In addition, increased NT has been associated with other pathologic conditions, including structural fetal abnormalities, cardiac malformations, a high risk of miscarriage, and intrauterine death. However, case-series reports have demonstrated that a substantial number of fetuses with increased NT during the first trimester have no anomalies and favorable neonatal outcomes.^[2,3]^

To assess this ultrasound marker, our study investigated the presence of increased NT in fetuses without chromosomal aneuploidies in an attempt to identify the proportions of other pathologies and normal fetuses associated with this marker.

## 2 Materials and methods

This study was conducted retrospectively on cases investigated by the Genetics Department of Iasi University of Medicine and Pharmacy Gr. T. Popa, Romania, between 2005 and 2014.

During this period, 2007 amniocenteses were performed for prenatal diagnosis due to various indications. In particular, increased NT was an indication for amniocentesis in 71 cases. NT was measured according to the criteria proposed by Nicolaides and reviewed by the Fetal Medical Foundation, with the 99th percentile defined to be 3.5 mm (irrespective of gestational age and/or crown-rump length).

The inclusion criteria for the study were:

- a measurement of >3.5 mm for NT, considered by initial standards as pathological
- a complete obstetrical record of the patient and the pregnancy that includes maternal age, gestational age, other associated abnormalities based on ultrasound reports, and serological markers for aneuploidy
- a description of the karyotype, by the FISH method, completed with cell culture karyotype
- the pregnancy outcome.

We excluded cases that did not satisfy all of the aforementioned criteria. In addition, we excluded cases for which data regarding the offspring outcome were unavailable.

- The genetic analysis was conducted via FISH for chromosomes 21, 13, 18, X and Y, and via karyotype cell culture.
- The results were entered into a database and analyzed in accordance with typical statistical assessment practices (Microsoft Excel, T Test evaluation).
- This article did not need approval from the Ethical Committee, as it was a retrospective one, and all patients had consented for their data to be used in clinical studies.

## 3 Results

In the 71 examined cases, the median maternal age was 30.5 years (range, 19–40 years), and the median gestational age at the time of amniocentesis was 17 weeks.

**Figure 1:**
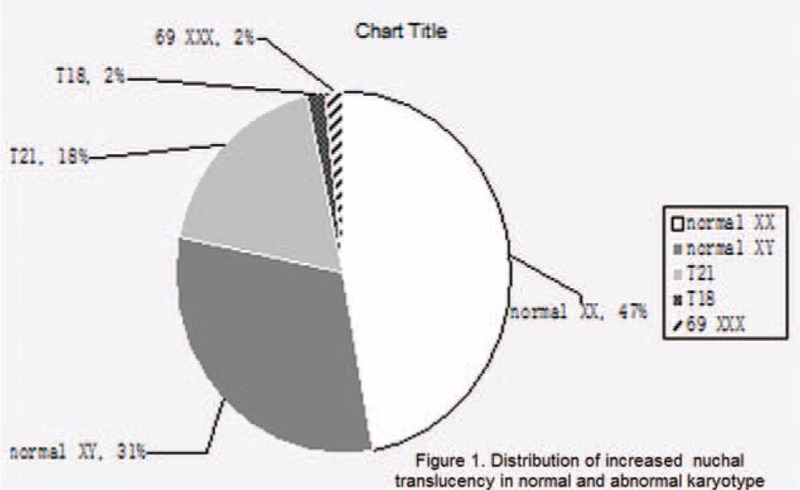
Normal and abnormal karyotypes.

Most of the selected cases (55 case, representing 78% of the total) had no aneuploidy with a normal 46 XX (33 cases, 47%) or 46 XY karyotype (22 cases, 31%) (Fig. 1). We could emphasize this as a comforting element for patients who could be stressed by the finding of an increased NT.

On the other hand, there were 13 cases (18.3%) of trisomy 21 (T21), 2 cases (2.8%) of trisomy 18 (T18), and 2 cases (2.8%) of triploidy.

**Figure 2:**
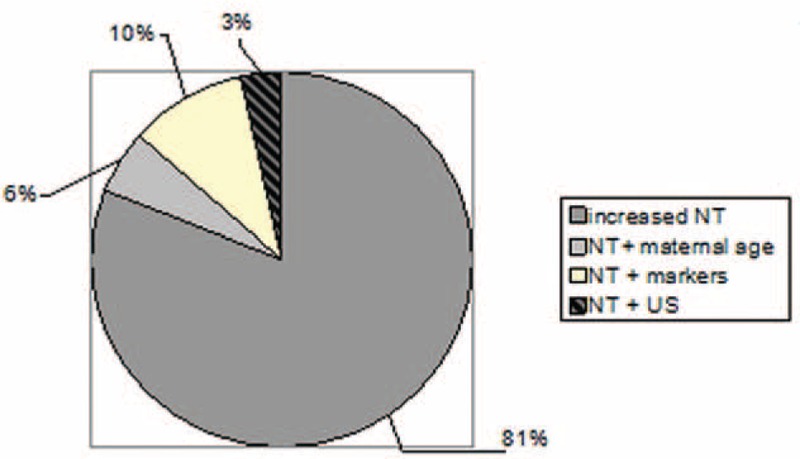
Associations of NT-indicated amniocentesis. NT = nuchal translucency.

In most of the 71 examined cases (81%), the indication for amniocentesis was increased NT alone; additional indications observed in the remaining cases included advanced maternal age (5.5%), abnormal serologic markers (10%), and other ultrasound findings (3.5%) (2 cases: heart abnormalities, fetal hydrops) (Fig. 2).

Pregnancy outcomes were evaluated for the 55 cases with normal karyotypes. A favorable outcome at term was obtained in 40 cases (72%); nonlethal cardiac or kidney conditions at birth or complications related to prematurity were observed in 6 cases, and miscarriages occurred in the remaining 6 cases. Three cases were lost at follow up and considered abnormal but excluded from diagnosed pathology.

## 4 Discussion

NT should be measured between 11 and 14 weeks of pregnancy (or for a crown-rump length of 45 to 84 mm), as recommended by Nicolaides et al^[1]^ in 2002. When the standards for NT measurement are appropriately followed, this marker could reduce the incidence of T21 at birth by 78%.

However, T21 is not the only aneuploidy associated with increased NT; for instance, Turner syndrome is also associated with increased NT. Different mechanisms appear to be involved; in T21, cardiac failure is thought to be the main driver of increased NT, whereas in Turner syndrome, increased NT results from abnormal or delayed lymphatic system development. In trisomic fetuses, the increased secretion of brain and atrial natriuretic hormone is another mechanism that could contribute to increased NT. In T18, a diaphragmatic hernia may impair venous return, resulting in increased NT. In our study, in addition to T21, 1 case of T18 and 1 case of triploidy were observed. Small chromosomal abnormalities at the subtelomeric level, which are extremely difficult to diagnose using FISH and cell culture Karyotype, may also have been present in certain cases.^[4]^

Euploid fetuses with increased NT may present with structural anomalies, including cardiac defects, diaphragmatic hernias, exomphalos, body stalk anomalies, and skeletal defects; certain genetic syndromes, such as congenital adrenal hyperplasia, fetal akinesia, or Noonan syndrome, have been cited as possible causes.^[5]^ A diaphragmatic hernia can occur in a fetus with a normal karyotype, as observed in a case described in a recent article.^[6]^ Other authors have reported a significant correlation between increased NT and a higher risk of orofacial cleft defects.^[7]^

An increased NT has also been associated with a high risk of miscarriage or fetal death. This risk increases with increasing NT thickness, and miscarriage or fetal death may be preceded by cardiac failure symptoms such as fetal hydrops. According to Goetzl,^[8]^ such miscarriages typically occur within the first 20 weeks of pregnancy.

In our study, indications for amniocentesis in our study were either increased NT alone or increased NT in combination with advanced maternal age, abnormal serum markers, or other ultrasound abnormalities. These indications have also been described in the literature, although prior studies have included additional analyses of increased NT. For instance, relative to female fetuses, male fetuses are more prone to increased NT but also have more favorable outcomes in cases involving increased NT (with an RR of 0.47 for adverse outcomes for males).^[9]^ This interesting finding is consistent with the results from our series, in which less favorable outcomes for euploid fetuses were more frequently observed among females than among males.

In addition, several studies emphasize the possibility that fetuses with NT>95th percentile may still have normal outcomes. The question is whether other factors, such as procedures like the intracytoplasmatic sperm injection (ICSI), are associated with a higher risk of increased NT but no other anomalies.^[10]^

In a large follow-up study, Vieira et al^[11]^ analyzed the outcomes of fetuses with increased NT and found that 54.4% of such fetuses were euploid and that 93% of these fetuses had normal births and immediate postnatal development, with only 3 cases involving miscarriage or neonatal death. In our study, outcomes were normal status at birth for 54% of fetuses, including 40 out of the 58 euploid cases (69%), and perinatal complications in 17% of cases. Regarding cases involving miscarriages or complications in the euploid group, there were 18 cases involving cardiac abnormalities, kidney failure, or complications due to prematurity (17% of all cases and 21% of euploid fetuses) and 6 miscarriages. Adverse outcomes were observed for 31% of euploid fetuses in our study, whereas in a study involving 87 women that used a similar methodology, the corresponding percentage was 39%. In this prior study, no correlation between increased NT and maternal age, fetal gender, or neurodevelopmentally delayed offspring was found.^[12]^

In our study, we were unable to follow the neurodevelopment of euploid children in cases involving increased NT. However, in other studies, the incidence of neurodevelopmental pathologies in children with increased NT, a normal karyotype, and normal anatomy did not appear to be higher than that reported for the general population.^[13]^

Our study has certain weaknesses related to the fact that prenatal scans and NT measurements were performed by different specialists during a time period when no clear standards such as those later provided by the Fetal Medical Foundation (FMF) recommendations had been proposed. To address these issues, we incorporated a cutoff value of 3.5 mm, which is accepted even by the FMF as indicative of a high probability of a true positive result. Also, our study used mainly FISH and cell culture karyotype to identify aneuploidies, as other methods were not available at that moment, for example Comparative genomic hybridization (CGH) array.

In conclusion, if aneuploidies are excluded, the management of increased NT in euploid fetuses should not greatly differ from the management of fetuses with normal NT. A thorough ultrasound examination performed at 20 to 24 weeks that should be interpreted in conjunction with other elements (clinical history and serum markers) is required. As De Domenico et al^[6]^ also emphasize in a review, the probabilities of delivering a baby with no major abnormalities are only approximately 70% for NT of 3.5 to 4.4 mm, 50% for NT of 4.5 to 5.4 mm, 30% for NT of 5.5 to 6.4 mm, and 15% for NT of 6.5 or more.
